# Dietary choline intake is necessary to prevent systems‐wide organ pathology and reduce Alzheimer's disease hallmarks

**DOI:** 10.1111/acel.13775

**Published:** 2023-01-15

**Authors:** Nikhil Dave, Jessica M. Judd, Annika Decker, Wendy Winslow, Patrick Sarette, Oscar Villarreal Espinosa, Savannah Tallino, Samantha K. Bartholomew, Alina Bilal, Jessica Sandler, Ian McDonough, Joanna K. Winstone, Erik A. Blackwood, Christopher Glembotski, Timothy Karr, Ramon Velazquez

**Affiliations:** ^1^ Arizona State University‐Banner Neurodegenerative Disease Research Center at the Biodesign Institute Arizona State University Tempe Arizona USA; ^2^ Arizona Alzheimer's Consortium Phoenix Arizona USA; ^3^ School of Life Sciences Arizona State University Tempe Arizona USA; ^4^ Translational Cardiovascular Research Center and Department of Internal Medicine University of Arizona College of Medicine Phoenix Arizona USA; ^5^ Biosciences Mass Spectrometry Facility, Biodesign Institute Arizona State University Tempe Arizona USA

**Keywords:** aging, aldob, amyloid‐beta, cardiac hypertrophy, choline, tau pathogenesis, liver, motor function

## Abstract

There is an urgent need to identify modifiable environmental risk factors that reduce the incidence of Alzheimer's disease (AD). The B‐like vitamin choline plays key roles in body‐ and brain‐related functions. Choline produced endogenously by the phosphatidylethanolamine N‐methyltransferase protein in the liver is not sufficient for adequate physiological functions, necessitating daily dietary intake. ~90% of Americans do not reach the recommended daily intake of dietary choline. Thus, it's imperative to determine whether dietary choline deficiency increases disease outcomes. Here, we placed 3xTg‐AD, a model of AD, and non‐transgenic (NonTg) control mice on either a standard laboratory diet with sufficient choline (ChN; 2.0 g/kg choline bitartrate) or a choline‐deficient diet (Ch‐; 0.0 g/kg choline bitartrate) from 3 to 12 (early to late adulthood) months of age. A Ch‐ diet reduced blood plasma choline levels, increased weight, and impaired both motor function and glucose metabolism in NonTg mice, with 3xTg‐AD mice showing greater deficits. Tissue analyses showed cardiac and liver pathology, elevated soluble and insoluble Amyloid‐β and Thioflavin S structures, and tau hyperphosphorylation at various pathological epitopes in the hippocampus and cortex of 3xTg‐AD Ch‐ mice. To gain mechanistic insight, we performed unbiased proteomics of hippocampal and blood plasma samples. Dietary choline deficiency altered hippocampal networks associated with microtubule function and postsynaptic membrane regulation. In plasma, dietary choline deficiency altered protein networks associated with insulin metabolism, mitochondrial function, inflammation, and fructose metabolic processing. Our data highlight that dietary choline intake is necessary to prevent systems‐wide organ pathology and reduce hallmark AD pathologies.

## Graphical abstract

Dietary choline deficiency throughout adulthood induces motor impairments, weight gain, impaired glucose metabolism, cardiac and liver pathology, and exacerbates Alzheimer's Disease (AD) hallmarks. If generalized to humans, these findings may help mitigate the estimated increase in the prevalence of AD and illustrates the importance of adequate dietary choline intake throughout adulthood to offset disease occurrence for the general population.

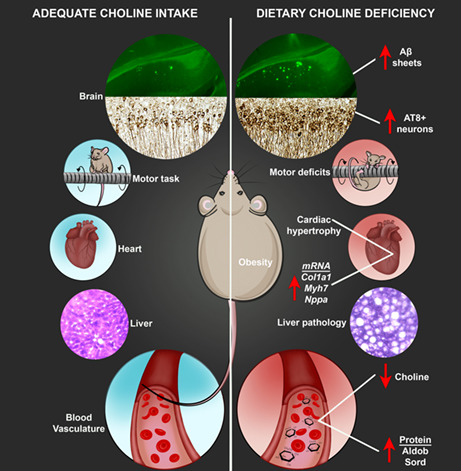

## 1 INTRODUCTION

Alzheimer's disease (AD) is one of the most prevalent age‐related neurodegenerative disorders in the US, with more than 6 million Americans currently living with the disease and a projected 16 million affected by 2050 (Alzheimer's Association, 2022). AD is characterized by two hallmark neuropathologies: extracellular amyloid‐beta (Aβ) plaques and intracellular neurofibrillary tangles (NFT), resulting in a progressive loss of cognitive abilities and memory (Deture & Dickson, 2019). The two major isoforms of Aβ are Aβ _40_ and Aβ_42_, found in both soluble and insoluble fractions (Deture & Dickson, 2019). These isoforms of Aβ aggregate into Aβ oligomers, which are neurotoxic precursors for insoluble Aβ plaques (Lee et al., 2017). Tau phosphorylated at serine 181 (pTau Ser181), pTau Ser396, and AT8 (Ser202/Threonine (Thr)205) are sites associated with tau pathogenesis in AD (Dave et al., 2021; Karikari et al., 2020; Mondragón‐Rodríguez et al., 2014). Other pathologies in AD include neuroinflammation (Husna Ibrahim et al., 2020; Kandimalla et al., 2017), cardiac pathology (Yang, Li, et al., 2020), and insulin dysregulation (Kandimalla et al., 2017). These physiological alterations indicate that AD is a complex, systems‐wide disease affecting several metabolic and cellular processes throughout the body. While a wealth of research has investigated these disease parameters separately, it remains unclear how exactly they contribute to AD pathogenesis or when they chronologically coincide with Aβ and NFT pathology. An abundance of work has highlighted that environmental factors may play a role in sporadic AD, which accounts for >95% of total cases (Kandimalla et al., 2017; Velazquez et al., 2019, 2020; Yang, Li, et al., 2020; Yang, Jiang, et al., 2020).

Choline, an essential nutrient found in a variety of foods, is a key precursor in the synthesis of choline phospholipids, betaine, and acetylcholine, a neurotransmitter involved in neurogenesis, synapse formation, learning, and memory (Blusztajn, 1998). Betaine is a methyl group donor in the conversion of homocysteine to methionine (Blusztajn, 1998), an amino acid that contributes to epigenetic regulation (Velazquez et al., 2020). Endogenous production of choline by phosphatidylethanolamine N‐methyltransferase (PEMT) in the livers of both mice and humans is not sufficient for normal metabolic functioning, requiring dietary intake (Institute of Medicine, 1998). In 1998, the Institute of Medicine (IOM) established an adequate daily intake (ADI) threshold for choline consumption to prevent fatty liver disease; 550 and 425 mg/day for adult men and women, respectively (Institute of Medicine, 1998). The ADI for pregnant women is 550 mg/day for healthy fetal development (Blusztajn, 1998; Institute of Medicine, 1998). However, recent studies show that ~90% of the US population fails to reach the ADI of choline (Zeisel, 2017).

Significant evidence shows that choline is important for healthy brain function (reviewed in Blusztajn, 1998; Zeisel, 2017). Maternal choline supplementation (MCS) of 4.5 times the ADI has been shown to produce important cognitive benefits for offspring in a mouse model of AD (Velazquez et al., 2020). Additionally, adulthood choline supplementation in a mouse model of AD significantly reduces Aβ plaque density, learning and memory deficits, and brain inflammation (Velazquez et al., 2019). Work has highlighted the relationship between choline and the dysfunction of systems‐wide cellular and molecular processes that are also implicated in AD. For example, choline supplementation is associated with attenuated microglial activation and reduced insulin resistance in the brain and periphery (Gao et al., 2017; Velazquez et al., 2019; Zeisel, 2017), and choline deficiency plays a role in cardiovascular disease, liver toxicity, and hypertension (Institute of Medicine, 1998; Millard et al., 2018); perturbations to these systems are known risk factors for AD, suggesting that choline deficiency could be a shared mechanism of these ailments. Moreover, work has shown that abnormalities in endogenous choline production via a functional single nucleotide polymorphism (rs7964) in the gene encoding PEMT are associated with increased AD incidence (Bi et al., 2012). Additionally, <100 mg of daily intake of choline is associated with an increased incidence of AD and dementia (Yuan et al., 2022). These studies suggest that reduced levels of choline may elevate the risk of AD, highlighting the importance of ADI.

Here, we sought to elucidate the effects of dietary choline deficiency in healthy aging and AD, placing NonTg and 3xTg‐AD mice (a mouse model of AD) on a choline deficient diet throughout adulthood. We hypothesized that a choline deficient diet will induce system‐wide cellular and molecular dysfunction throughout the body and brain, increasing the risk of AD across several pathogenic axes.

## 2 RESULTS

**FIGURE 1:**
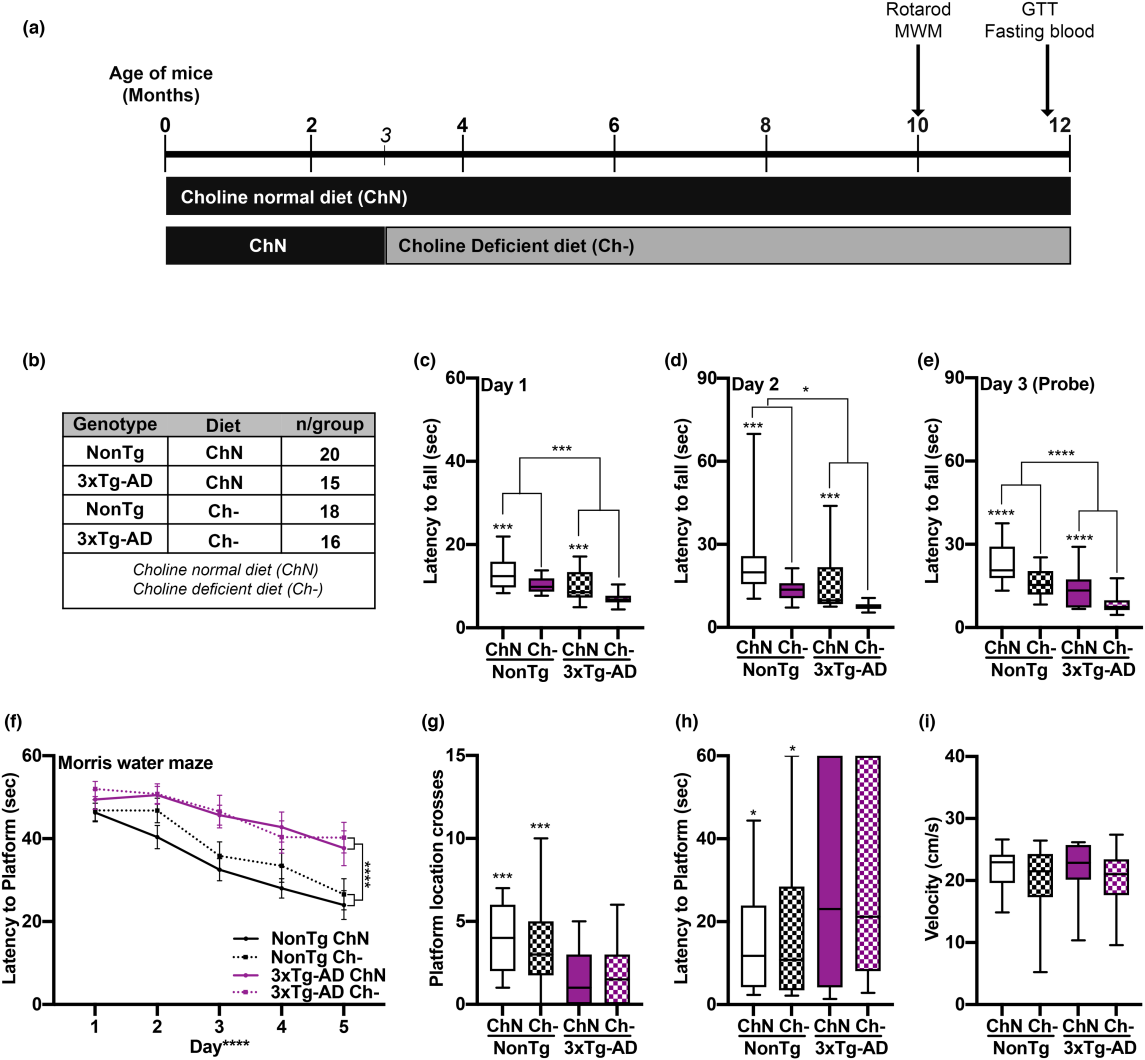
Ch‐ impairs motor function in both NonTg and 3xTg‐AD mice. (a) Experimental design. (b) Groups and sample sizes. (c‐e) Motor function assessment via the rotarod task illustrated significant main effects of genotype, where the 3xTg‐AD mice fell off the spinning rod significantly faster than NonTg mice on Day 1 (*p* = 0.001), Day 2 (*p* = 0.016) and the Day 3 probe trial (*p* < 0.0001). The assessment also showed significant main effects of diet, where the Ch‐ mice fell significantly faster on Day 1 (*p* = 0.0002), Day 2 (*p* = 0.0002), and Day 3 (*p* < 0.0001), than their ChN counterparts. (f) Morris water maze testing revealed significant effects of genotype, where the 3xTg‐AD had a higher latency to find the platform across the 5 days of learning than the NonTg mice (*p* < 0.0001). (g‐i) During the Day 6 probe trial, the 3xTg‐AD mice crossed the platform significantly fewer times (*p* = 0.0008) and had a higher latency to first cross the platform location (*p* = 0.0105) than the NonTg mice. Swim speed was equal across the groups. For box plots, the center line represents the median value, the limits represent the 25th and 75th percentile, and the whiskers represent the minimum and maximum values of the distribution. Line graphs are mean ± SE. *p < 0.05, ***p < 0.001, ****p < 0.0001

At 3 months of age, we exposed female NonTg and 3xTg‐AD mice to either a control choline (ChN; 2.0 g/kg) or choline‐deficient (Ch‐; choline deficiency; 0.0 g/kg; Figure S1a,b) diet, producing four experimental groups (NonTg ChN, *n* = 20; 3xTg‐AD ChN, *n* = 15; NonTg Ch‐, *n* = 18; 3xTg‐AD Ch‐, *n* = 16; Figure 1b). Mice were aged to 10 months and tested in the rotarod task to assess motor function, and the Morris water maze (MWM) to assess spatial reference learning and memory (Figure 1a). Blood and tissues were subsequently collected for pathological and proteomic assessment.

### 2.1 Ch‐ throughout adulthood impairs motor function

For the rotarod task, during the two training days, we found significant main effects of genotype and diet for latency to fall off the spinning rod on Day 1 (*F*_(1, 65)_ = 16.72, *p* = 0.001; Figure 1c), (*F*_(1, 65)_ = 15.86, *p* = 0.0002) and Day 2 (*F*_(1, 65)_ = 6.114, *p* = 0.016; Figure 1d), (*F*_(1, 65)_ = 15.69, *p* = 0.0002), where 3xTg‐AD mice fell sooner than NonTg mice, and Ch‐ mice fell sooner than ChN mice. On probe Day 3, we found significant main effects of genotype (*F*_(1, 65)_ = 20.99, *p* < 0.0001; Figure 1e) and diet (*F*_(1, 65)_ = 20.56, *p* < 0.0001), where 3xTg‐AD mice fell sooner than NonTg mice, and Ch‐ mice fell sooner than the ChN mice, indicating that the AD phenotype and Ch‐ impaired motor function.

Mice were then tested in the MWM for 6 consecutive days. One 3xTg‐AD Ch‐ mouse was excluded for inability to swim. During the first 5 training days, mice received four trials/day. For latency to find the hidden platform, we found a significant main effect of day, indicating learning (*F*_(1, 256)_ = 26.932 *p* < 0.0001: Figure 1f). We also found a significant main effect of genotype (*F*_(1, 256)_ = 23.335, *p* < 0.0001), where 3xTg‐AD mice took significantly longer to find the hidden platform than NonTg mice. No significant diet main effects or interactions were found. On Day 6, the platform was removed, and mice were tested in a 60‐s probe trial to assess spatial memory. 3xTg‐AD mice crossed the platform location significantly fewer times than NonTg mice (*F*_(1, 64)_ = 12.276, *p* = 0.0008; Figure 1g) and had a higher latency to first cross the platform location (*F*_(1, 64)_ = 6.948, *p* = 0.0105; Figure 1h), illustrating deficits. No diet effects were detected. Swim speed during the probe trials was similar across groups (Figure 1i). These data indicate that Ch‐ does not exacerbate spatial memory impairments in 3xTg‐AD mice nor impair performance in NonTg mice.

### 2.2 Ch‐ increases body weight, impairs glucose metabolism, and reduces plasma choline levels

**FIGURE 2:**
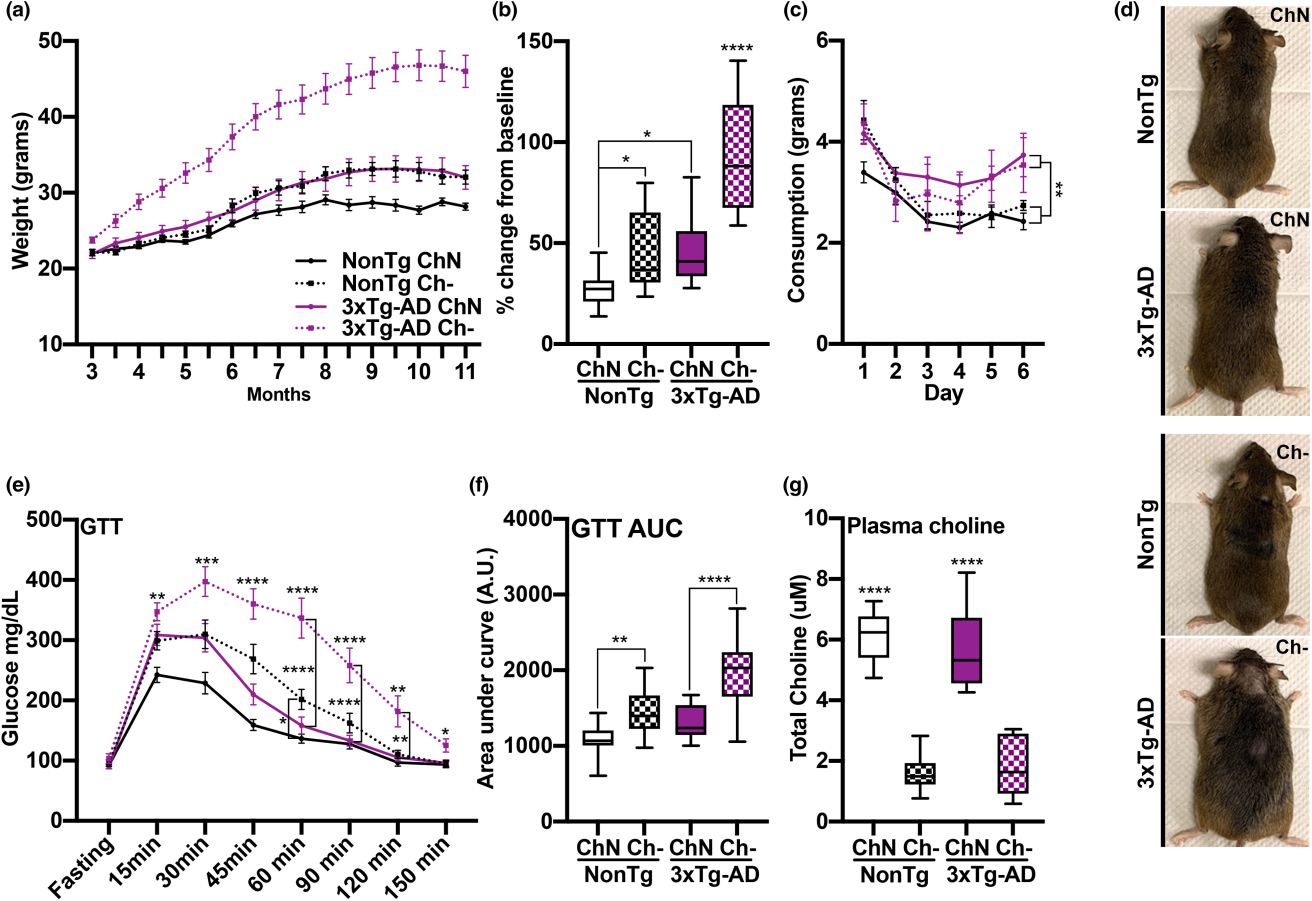
Ch‐ increases body weight, impairs glucose metabolism, and reduces plasma choline levels. (a) Body weight across age. (b) Percent weight change from baseline shows elevated levels in the NonTg Ch‐ mice compared to NonTg ChN mice (*p* = 0.026) and in the 3xTg‐AD Ch‐ mice compared to 3xTg‐AD ChN mice (*p* < 0.0001). (c) 3xTg‐AD mice had significantly higher food intake across the 6 days of the food consumption test than the NonTg mice (*p* = 0.0042), but no diet effect was detected. (d) Representative images of mice illustrating weight differences. (e) Ch‐ mice had significantly higher glucose levels in the glucose tolerance test (GTT) than ChN mice (*p* < 0.05). 3xTg‐AD Ch‐ mice had significantly higher glucose levels in the GTT from the 60 min through 120 min timepoints than their ChN counterparts (*p* < 0.01). NonTg Ch‐ mice had significantly higher glucose levels than their ChN counterparts at the 60 min time point (*p* < 0.05). (f) GTT area under the curve (AUC) analysis showed higher levels in NonTg Ch‐ mice than their ChN counterparts (*p* = 0.0042), and 3xTg‐AD Ch‐ mice had higher levels than their ChN counterparts (*p* < 0.0001). (g) Ch‐ significantly reduced plasma choline levels (*p* < 0.0001). Line graphs are mean ± SE. For box plots, the center line represents the median value the limits represent the 25th and 75th percentile, and the whiskers represent the minimum and maximum value of the distribution. *p < 0.05, **p < 0.01, ***p < 0.001, ****p < 0.0001

Choline plays an essential role in glucose metabolism, a biological system that is dysfunctional in AD (Gao et al., 2017; Millard et al., 2018). We examined body weight and performed a glucose tolerance test (GTT). For percent weight change from baseline, we found significant main effects of genotype (*F*_(1, 65)_ = 51.96, *p* < 0.0001) and diet (*F*_(1,65)_ = 52.86, *p* < 0.0001), where 3xTg‐AD mice had a higher percent weight change than NonTg mice (Figure 2a,b). Ch‐ mice had a higher percent weight change than their ChN counterparts. We also found a significant genotype by diet interaction (*F*_(1, 65)_ = 10.91, *p* = 0.0016), where NonTg Ch‐ mice had a higher percent weight change from baseline than NonTg ChN mice (*p* = 0.026). 3xTg‐AD Ch‐ mice showed a higher percentage weight change from baseline than 3xTg‐AD ChN mice (*p* < 0.0001). Notably, NonTg Ch‐ and 3xTg‐AD ChN mice showed no significant difference (*p* > 0.99), indicating that the weight gain in NonTg Ch‐ mice phenocopies that seen in the AD mouse. To determine if body weight differences arose from food intake, we performed a food consumption test. We found a significant main effect of genotype (*F*_(1, 14)_ = 11.69, *p* = 0.0042; Figure 2c), where the 3xTg‐AD mice consumed significantly more than NonTg mice, but no significant diet effects, illustrating that body weight differences between diets (Figure 2d) are not due to food consumption.

In the GTT, we found significant main effects of genotype (*F*_(1, 65)_ = 26.02, *p* < 0.0001) and diet (*F*_(1, 65)_ = 47.01, *p* < 0.0001), where 3xTg‐AD mice show higher glucose levels than NonTg mice. Ch‐ mice had higher glucose levels than ChN mice (Figure 2e). We also found a significant genotype by diet by time interaction (*F*_(7,455)_ = 2.674, *p* = 0.0101), where 3xTg‐AD Ch‐ mice had significantly higher glucose levels from the 60 min through 120 min time points compared to 3xTg‐AD ChN mice (*p* < 0.01). NonTg Ch‐ mice had significantly higher glucose levels than NonTg ChN mice at the 60 min time point (*p* < 0.05). Lastly, we analyzed the glucose area under the curve (AUC), as it provides a better assessment of glucose tolerance (Velazquez et al., 2017). We found significant main effects of genotype (*F*_(1, 65)_ = 26.04, *p* < 0.0001) and diet (*F*_(1, 65)_ = 47.25, *p* < 0.0001), where the glucose AUC for 3xTg‐AD mice was higher than for NonTg mice and higher for Ch‐ mice than for ChN mice (Figure 2f). We also found a significant genotype by diet interaction (*F*_(1, 65)_ = 4.393, *p* = 0.040), where AUC was higher in NonTg Ch‐ than NonTg ChN mice (*p* = 0.0042), and in 3xTg‐AD Ch‐ compared to 3xTg‐AD ChN mice (*p* < 0.0001). AUC was similar between the NonTg Ch‐ and 3xTg‐AD ChN mice (*p* > 0.9999). Collectively, these results show that Ch‐ increases weight and impairs glucose metabolism in both the control and AD mice, with 3xTg‐AD mice showing greater deficits. This is notable because weight gain and impaired glucose metabolism are risk factors for AD (Velazquez et al., 2017), highlighting the importance of dietary choline to deter metabolic deficits.

To determine whether choline levels were affected by diet, we collected blood prior to euthanasia and isolated plasma. We found a significant main effect of diet (*F*_(1, 20)_ = 96.08, *p* < 0.0001), where Ch‐ mice (*n* = 6/group) had significantly lower levels of circulating choline in their plasma than ChN mice (Figure 2g).

### 2.3 Ch‐ induces cardiac and liver pathology

**FIGURE 3:**
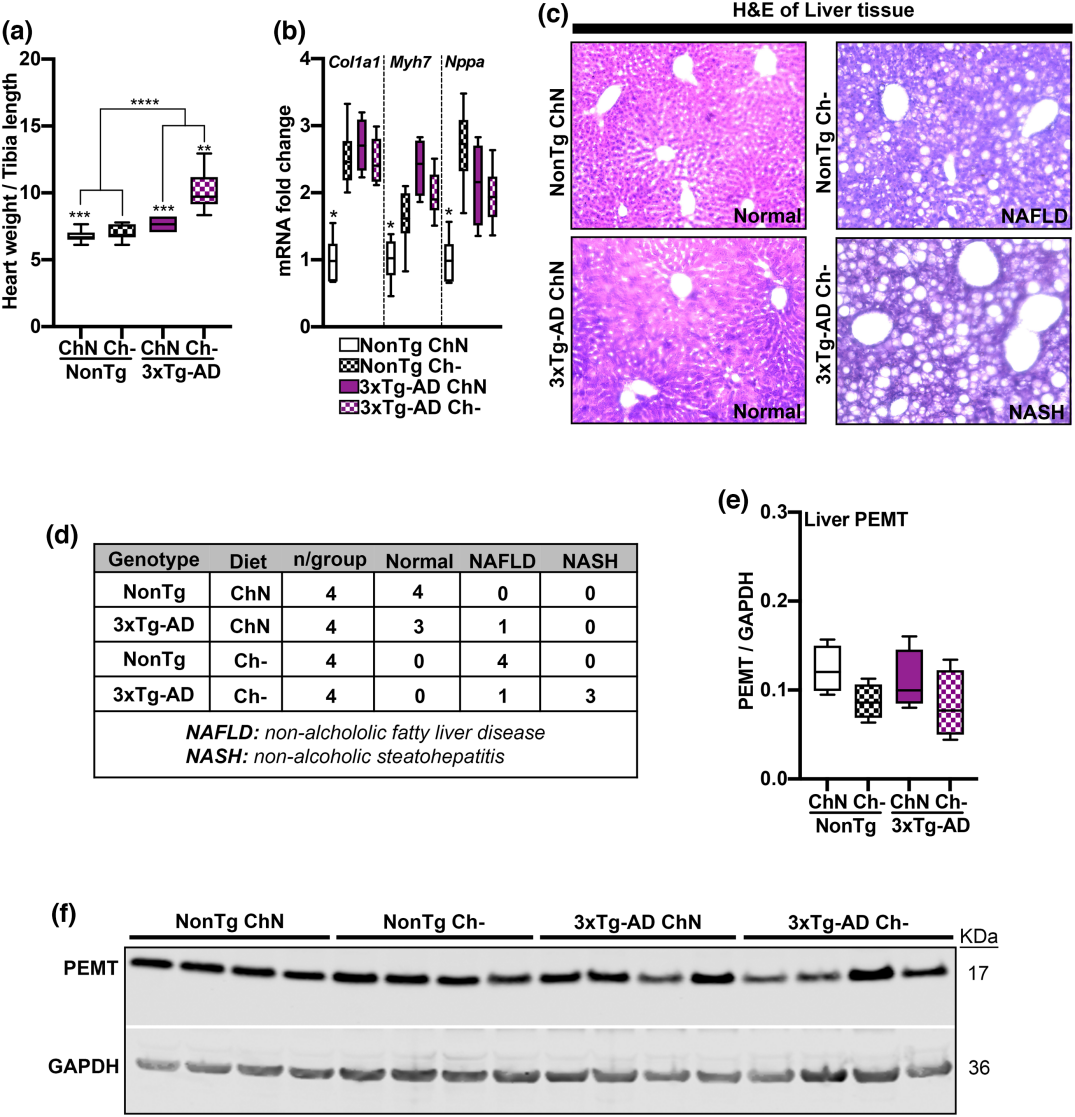
Ch‐ induces cardiac and liver pathology. (a) 3xTg‐AD mice had a higher heart weight than NonTg mice (*p* < 0.0001), and the Ch‐ groups had higher heart weights than the ChN mice (*p* = 0.001). 3xTg‐AD Ch‐ mice had the highest heart weight (*p* = 0.0018). (b) mRNA expression levels of *Col1a1*, *Myh7*, and *Nppa* were elevated in 3xTg‐AD ChN mice and both NonTg and 3xTg‐AD Ch‐ mice compared to the NonTg ChN group (*p* < 0.05). (c) Photomicrographs of liver tissue stained with Hematoxylin and Eosin (H&E). (d) Table reporting liver pathology in Ch‐ mice. (e,f) Western blot and quantification for PEMT illustrate no differences across groups. For box plots, the center line represents the median value the limits represent the 25th and 75th percentile, and the whiskers represent the minimum and maximum values of the distribution. *p < 0.05, **p < 0.01, ***p < 0.001, ****p < 0.0001

Given the role of choline and AD in cardiac dysfunction (Millard et al., 2018; Yang, Li, et al., 2020; Yang, Jiang, et al., 2020), we examined cardiac pathology. We first assessed heart weight normalized by tibia length as a measure of pathological cardiac hypertrophy (Long et al., 2022); NonTg ChN (*n* = 9), NonTg Ch‐ (*n* = 7), 3xTg‐AD ChN (*n* = 4), and 3xTg‐AD Ch‐ (*n* = 6). We found significant main effects of genotype (*F*_(1, 22)_ = 29.63, *p* < 0.0001) and diet (*F*_(1, 22)_ = 13.78, *p* = 0.001), where 3xTg‐AD mice had a higher heart weight than NonTg mice (Figure 3a). Ch‐ groups had higher heart weights than ChN mice. We also found a significant genotype by diet interaction (*F*_(1, 22)_ = 13.78, *p* = 0.001), where 3xTg‐AD Ch‐ mice had higher heart weights than all other groups (*p* = 0.0018). Next, total RNA was extracted from snap‐frozen left ventricular extracts and subjected to qRT‐PCR for transcript analysis; NonTg ChN (*n* = 6), NonTg Ch‐ (*n* = 6), 3xTg‐AD ChN (*n* = 4), and 3xTg‐AD Ch‐ (*n* = 6). We examined the expression levels of *Col1a1*, *Myh7*, and *Nppa*, which are genes whose increased expression is associated with cardiac pathology (Hua et al., 2020; Man et al., 2018; Montag et al., 2018). We found significant genotype by diet interactions ((*F*_(1, 18)_ = 27.85, *p* < 0.0001; Figure 3b), (*F*_(1, 18)_ = 9.743, *p* = 0.006), (*F*_(1, 18)_ = 19.17, *p* = 0.0004), respectively) revealing that NonTg ChN mice had lower expression of *Col1a1*, *Myh7*, and *Nppa* than all other groups (*p* < 0.05). These data demonstrate that cardiac dysfunction can be caused by both AD mutations and Ch‐.

The existing guidelines for dietary choline established in 1998 by the IOM were developed to prevent non‐alcoholic fatty liver disease (NAFLD; Institute of Medicine, 1998). To determine if adulthood Ch‐ induced liver disease, we assessed pathology (*n* = 4 mice/group) using a Hematoxylin and Eosin (H&E) stain (Figure 3c). Staining for the ChN mice showed that NonTg mice had healthy livers and one 3xTg‐AD mouse showed evidence of NAFLD (Figure 3d). All the NonTg Ch‐ mice exhibited NAFLD, and three 3xTg‐AD Ch‐ mice showed non‐alcoholic steatohepatitis (NASH). We measured protein levels of PEMT from liver tissue to determine if Ch‐ led to compensatory upregulation. We found no significant differences across the four groups (Figure 3e, f). Collectively, these results show that a Ch‐ diet induces pathology in multiple organ systems associated with metabolic function.

### 2.4 Ch‐ reduces hippocampal (Hp) and cortical (Ctx) choline levels and exacerbates soluble and insoluble amyloid‐ß (Aß) pathology and tau hyperphosphorylation

**FIGURE 4:**
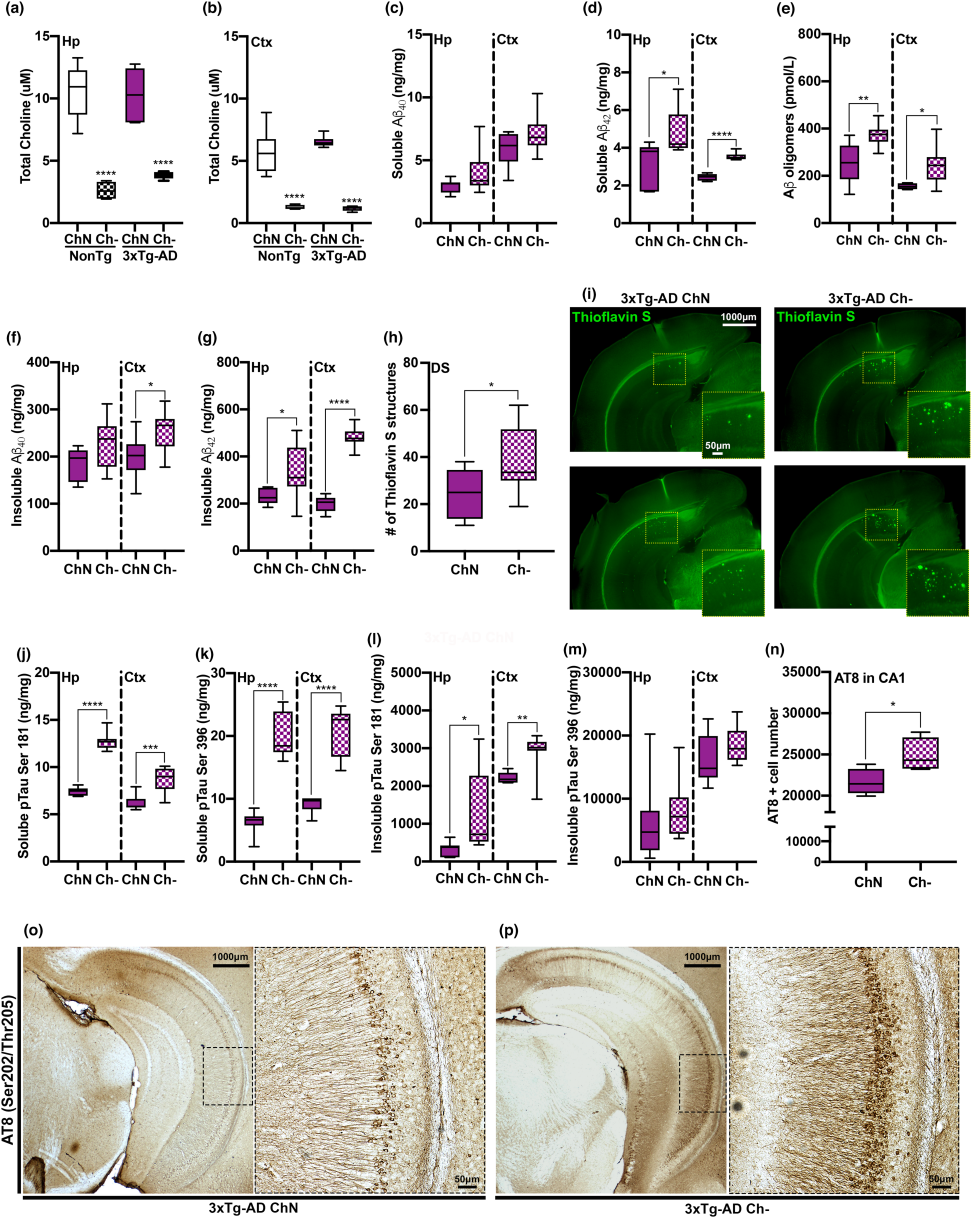
Ch‐ reduces hippocampal (Hp) and cortical (Ctx) choline levels and exacerbates soluble and insoluble amyloid‐β (Aβ) fractions and tau phosphorylation of pathological epitopes. (a, b) Hp (*p* < 0.0001) and Ctx (*p* < 0.0001) choline levels were significantly reduced in the Ch‐ mice compared to their ChN counterparts. NonTg mice do not display Aβ pathology and were therefore excluded from Aβ analyses. (c) No significant differences were detected in soluble Aβ_40_. (d) Soluble Aβ_42_ levels were significantly elevated in the Hp (*p* = 0.014) and Ctx (*p* < 0.0001) of 3xTg‐AD Ch‐ mice. (e) Aβ oligomer levels were significantly elevated in the Hp (*p* = 0.0054) and Ctx (*p* = 0.021) of 3xTg‐AD Ch‐ mice. (f) For insoluble Aβ_40_ levels, we found a non‐significant trend in the Hp (*p* = 0.077) and significantly elevated levels in the Ctx (*p* = 0.032) of 3xTg‐AD Ch‐ mice. (g) Insoluble Aβ_42_ levels were significantly elevated in the Hp (*p* = 0.035) and Ctx (*p* < 0.0001) of 3xTg‐AD Ch‐ mice. (h) Thioflavin S Aβ structure count was significantly higher in the dorsal subiculum (DS) of 3xTg‐AD Ch‐ mice. (i) Photomicrographs of Thioflavin S staining, two representative images per 3xTg‐AD group. (j) Soluble levels of phosphorylated tau (pTau) at serine (Ser)181 were significantly elevated in the Hp (*p* < 0.0001) and Ctx (*p* = 0.0009) of 3xTg‐AD Ch‐ mice. (k) Soluble levels of pTau at Ser396 were significantly elevated in the Hp (*p* < 0.0001) and Ctx (*p* < 0.0001) of 3xTg‐AD Ch‐ mice. (l,m) Insoluble levels of pTau at Ser181 were significantly elevated in the Hp (*p* = 0.0442) and Ctx (*p* = 0.0052) of 3xTg‐AD Ch‐ mice. No significant difference was observed for insoluble pTau at Ser396 in the Hp and Ctx. (n) AT8 (Ser202/Threonine (Thr)205)‐positive cell numbers were significantly elevated in the CA1 region of the Hp of 3xTg‐AD Ch‐ mice (*p* = 0.049). (o,p) Photomicrographs of ventral Hp sections stained for AT8 at 2.5× and 10×. For box plots, the center line represents the median value, the limits represent the 25th and 75th percentile, and the whiskers represent the minimum and maximum value of the distribution. *p < 0.05, **p < 0.01, ***p < 0.001, ****p < 0.0001

We measured Hp and Ctx (*n* = 6/group) levels of choline to determine the effects of dietary Ch‐. Brain choline levels rise at a predicable rate at post‐mortem (Ceder & Schuberth, 1977), thus comparisons between choline plasma levels from live animals and brain levels taken post‐mortem should be made with caution. We found a significant main effect of diet for both Hp (*F*_(1, 20)_ = 133.50, *p* < 0.0001) and Ctx (*F*_(1, 20)_ = 165.30, *p* < 0.0001) choline levels, where Ch‐ mice had significantly lower levels than ChN mice (Figure 4a,b). To understand the effects of Ch‐ on AD pathogenesis, we used ELISAs to quantify soluble and insoluble Aβ in 3xTg‐AD Ch‐ (*n* = 8) and ChN (*n* = 7) mice. NonTg mice do not display Aβ or tau pathology and therefore were excluded from these analyses (Velazquez et al., 2019, 2020). For soluble Aβ_40_ fractions, we found no significant differences in Hp (t_(13)_ = 1.712, *p* = 0.111) or Ctx (t_(13)_ = 1.685, *p* = 0.116) levels between 3xTg‐AD mice (Figure 4c). For soluble Aβ_42,_ we found that 3xTg‐AD Ch‐ mice had higher levels in the Hp (t_(13)_ = 2.833, *p* = 0.014) and Ctx (t_(13)_ = 11.59, *p* < 0.0001; Figure 4d). Similarly, for soluble Aβ oligomers, 3xTg‐AD Ch‐ mice had significantly higher levels in the Hp (t_(13)_ = 3.335, *p* = 0.0054) and Ctx (t_(13)_ = 2.648, *p* = 0.021; Figure 4e). For insoluble Aβ_40_ fractions in the Hp, we found a non‐significant trend where 3xTg‐AD Ch‐ had higher levels than ChN mice (t_(13)_ = 1.919, *p* = 0.077; Figure 4f). In the Ctx (t_(13)_ = 2.398, *p* = 0.032), 3xTg‐AD Ch‐ mice exhibited significantly higher levels of insoluble Aβ_40_ (Figure 4f). For insoluble Aβ_42_ fractions, 3xTg‐AD Ch‐ mice had significantly higher levels in the Hp (t_(13)_ = 2.357, *p* = 0.035) and Ctx (t_(13)_ = 13.90, *p* < 0.0001; Figure 4g). Lastly, we stained tissue for Thioflavin S to quantify Aβ sheets in the dorsal subiculum (DS) of the Hp of 3xTg‐AD mice (*n* = 4 mice/group, 2 sections per animal). We found a significantly higher count in 3xTg‐AD Ch‐ mice (t_(14)_ = 2.281, *p* = 0.039; Figure 4h,i), collectively showing that Ch‐ increases levels of toxic Aβ pathology in 3xTg‐AD mice.

We also sought to understand the effects of Ch‐ on tau pathogenesis via ELISAs to detect soluble and insoluble fractions of phosphorylated tau (pTau) in 3xTg‐AD mice. For soluble pTau Ser181, we found significantly higher levels in the Hp (t_(13)_ = 13.92, *p* < 0.0001) and Ctx (t_(13)_ = 4.293, *p* = 0.0009) of 3xTg‐AD Ch‐ mice (Figure 4j). For soluble pTau Ser396, we found significantly higher levels in the Hp (t_(13)_ = 9.045, *p* < 0.0001) and Ctx (t_(13)_ = 7.691, *p* < 0.0001) in 3xTg‐AD Ch‐ mice (Figure 4k). For insoluble pTau Ser181, we found significantly higher levels in the Hp (t_(13)_ = 2.228, *p* = 0.0442) and Ctx (t_(13)_ = 3.356, *p* = 0.0052) in 3xTg‐AD Ch‐ mice (Figure 4l). No significant differences between 3xTg‐AD ChN and Ch‐ mice were detected for insoluble pTau Ser396 in Hp (t_(13)_ = 0.622, *p* = 0.545) or Ctx (t_(13)_ = 1.436, *p* = 0.175) tissue (Figure 4m). Lastly, we stained against AT8, which is associated with intraneuronal tau filaments (Dave et al., 2021), and found significantly higher stained cells in 3xTg‐AD Ch‐ mice (t_(6)_ = 2.462, *p* = 0.049; Figure 4n–p). Collectively, these data illustrate that Ch‐ exacerbates tau pathology in 3xTg‐AD mice.

### 2.5 3xTg‐AD ChN mice show protein abundance changes in Hp proteins related to AD

To further understand how Ch‐ altered protein networks in the Hp, we performed LC–MS/MS coupled with LFQ (*n* = 4/group) and identified 3544 proteins (Supplemental Figure S2). The threshold for proteins considered to be differentially abundant for all analysis is adj. *p*‐value < 0.05, −1 > Log_2_ FC > 1. We first compared the Hp proteome of 3xTg‐AD ChN vs. NonTg ChN mice (Figure S2 and Table S1) and found 46 differentially abundant proteins, 19 of which were upregulated, and 27 of which were downregulated. We found well‐known AD‐associated proteins altered by the 3xTg‐AD genotype, including upregulation of the amyloid precursor protein (App; Log_2_ FC = 1.58) and microtubule‐associated protein tau (Mapt), although Log_2_ FC was just under 1 (Log_2_ FC = 0.97), as well as downregulation of Apolipoprotein A1 (Apoa1; Log_2_ FC = −1.27), and Neuroplastin (Nptn; Log_2_ FC = −1.55). App is involved in the production of Aβ, while Mapt is associated with microtubule stability and its gain‐of‐function leads to tau pathogenesis (Deture & Dickson, 2019; Velazquez et al., 2018, 2017). Apoa1 is the major protein component of the high‐density lipoprotein involved in cholesterol transport, and its downregulation is implicated in AD. Nptn is a critical protein for long‐term potentiation at Hp excitatory synapses linked to AD (Deture & Dickson, 2019; Ilic et al., 2019). These findings support that the transgenes in the 3xTg‐AD model result in a phenotype consistent with human AD (Deture & Dickson, 2019).

### 2.6 Ch‐ alters Hp protein networks associated with metabolic processing in NonTg mice and microtubule function and postsynaptic membrane regulation in 3xTg‐AD mice

**FIGURE 5:**
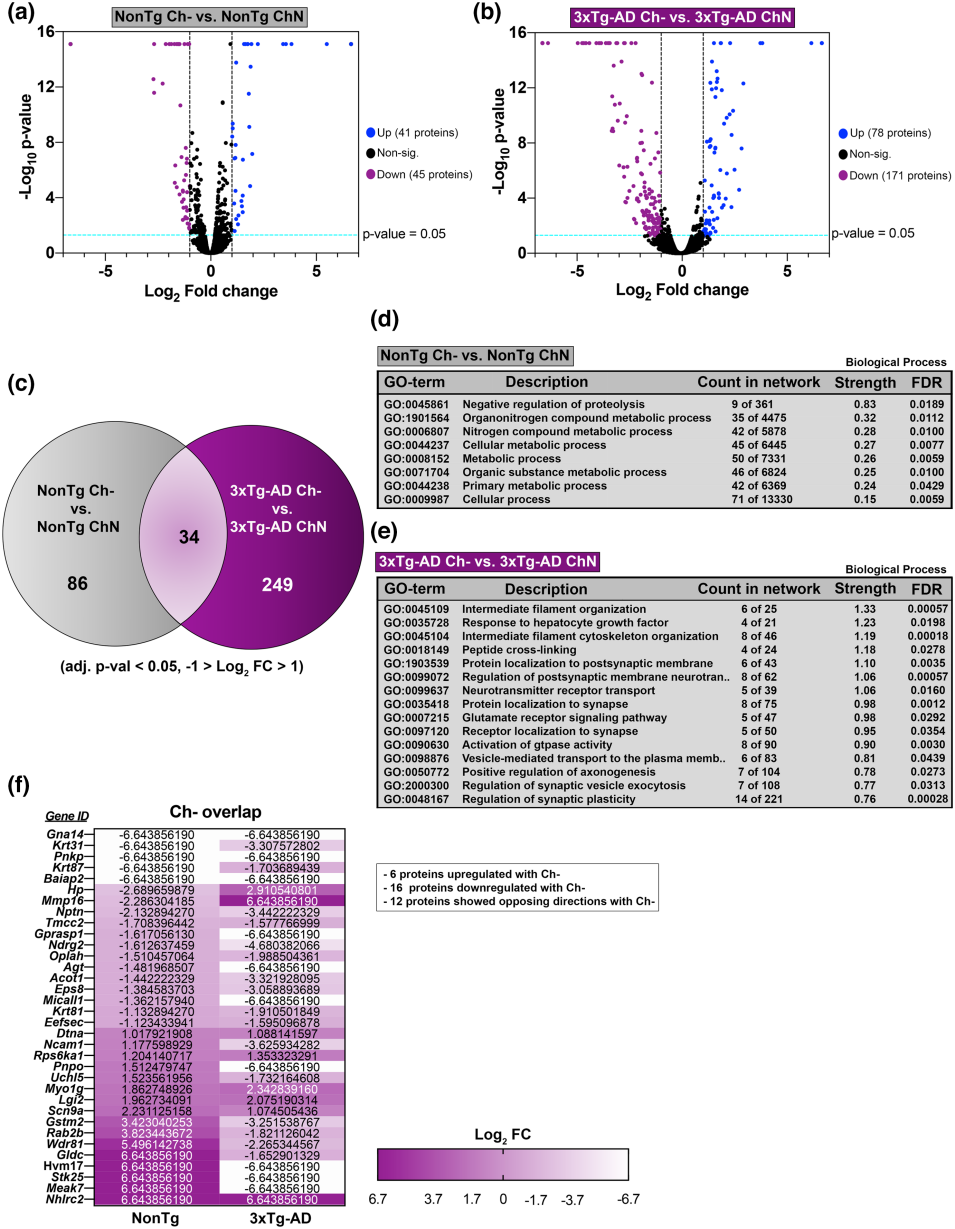
Ch‐ alters Hp protein networks in both NonTg and 3xTg‐AD mice. (a,b) Liquid chromatography‐tandem mass spectrometry followed by label‐free quantification (adj. *p*‐value < 0.05, −1 > Log_2_ FC >1) identified 86 differentially abundant proteins between NonTg Ch‐ and NonTg ChN Hp, and 249 differentially abundant proteins between 3xTg‐AD Ch‐ and 3xTg‐AD ChN Hp. (c) Thirty‐four proteins were commonly identified as differentially abundant due to Ch‐ in both NonTg and 3xTg‐AD Hp. (d,e) NonTg Ch‐ vs. NonTg ChN and 3xTg‐AD Ch‐ vs. 3xTg‐ AD ChN gene ontology (GO) biological process classification analyses (for (e), the top 15 biological processes are displayed based on strength of prediction). (f) List of genes and Log_2_ fold changes corresponding to the 34 proteins overlapping due to Ch‐

We compared the Hp proteomes of NonTg Ch‐ vs. NonTg ChN mice and 3xTg‐AD Ch‐ vs. 3xTg‐AD ChN mice (Figure S2). In the NonTg Ch‐ vs. NonTg ChN comparison, 86 differentially abundant proteins were identified, and in the 3xTg‐AD Ch‐ vs. 3xTg‐AD ChN comparison, 249 differentially abundant proteins were identified (Figure 5a,b). Thirty‐four proteins were commonly identified as differentially abundant due to Ch‐ in both NonTg and 3xTg‐AD mice (Figure 5c; Figure S2). Next, we performed gene ontology (GO) on the three sets of differentially abundant proteins to understand what biological processes and molecular functions were represented in these data sets. In the NonTg Ch‐ vs. NonTg ChN comparison, GO revealed changes in pathways associated with the organonitrogen compound metabolic process, nitrogen compound metabolic process, and cellular metabolic process (Figure 5d; full GO Figure S3), corroborating choline's well‐documented role in metabolic processing (Gao et al., 2017).

In the 3xTg‐AD Ch‐ vs. 3xTg‐AD ChN comparison, GO revealed changes in protein networks closely associated with AD pathology (Figure 5e; full GO Figure S3). Significant biological processes included intermediate filament cytoskeleton organization, cytoskeleton organization, regulation of vesicle‐mediated transport to the plasma membrane, and vesicle‐mediated transport in the synapse. These pathways are notable because they are directly related to microtubule function, which is closely tied to tau pathology in AD (Deture & Dickson, 2019). Hyperphosphorylation of tau results in its disassociation from microtubules, disrupting microtubule stability, and axonal trafficking (Deture & Dickson, 2019). This data suggests that Ch‐ may alter microtubule function in 3xTg‐AD Hp and exacerbate microtubule dysfunction caused by tau pathogenesis. GO also revealed a series of significant biological processes associated with postsynaptic membrane regulation including protein localization to postsynaptic membrane, regulation of postsynaptic membrane neurotransmitter receptors, neurotransmitter receptor transport, protein localization to the synapse, receptor localization to the synapse, regulation of synaptic vesicle exocytosis, and regulation of synaptic plasticity. These findings corroborate previous work showing that adulthood dietary choline supplementation modulates the abundance of critical neuroreceptors in the 3xTg‐AD Hp (Velazquez et al., 2019).

Of the 34 proteins that were differentially abundant due to Ch‐ in both NonTg Ch‐ vs. NonTg ChN and 3xTg‐AD Ch‐ vs. 3xTg‐AD ChN comparisons (Figure 5f; Figure S2), 6 were upregulated, 16 were downregulated, and 12 were expressed in opposing directions. GO revealed that no biological processes or molecular functions were represented by these 34 proteins.

### 2.7 3xTg‐AD ChN mice show protein abundance changes in plasma proteins related to AD

To understand the mechanisms by which Ch‐ may contribute to systems‐wide dysfunction, we performed LC–MS/MS coupled with LFQ (*n* = 4/group) of the plasma proteome and identified 734 proteins (Figure S2). We first compared the plasma proteome of 3xTg‐AD ChN vs. NonTg ChN mice (Figure S2 and Table 2), and found 126 differentially abundant proteins, 49 of which were upregulated and 76 downregulated. Notably, we found alterations in levels of proteins that are well‐known to be associated with the AD phenotype, including downregulation of serum amyloid (Sa)a1 (Log_2_ FC = −2.45), Saa2 (Log_2_ FC = −2.51), and upregulation of voltage‐dependent anion channel (Vdac) 1 (Log_2_ FC = 2.38), Vdac2 (Log_2_ FC = 6.64), insulin‐degrading enzyme (Ide; Log_2_ FC = 2.57), and heat shock protein family D (Hspd) 1 (Log_2_ FC = 6.64) (Jang et al., 2019; Kandimalla et al., 2017; Shoshan‐Barmatz et al., 2018; Singulani et al., 2020), indicating that the 3xTg‐AD model displays a plasma phenotype that is representative of human AD (Deture & Dickson, 2019).

### 2.8 Ch‐ alters plasma protein networks associated with immune response and inflammation in NonTg mice

**FIGURE 6:**
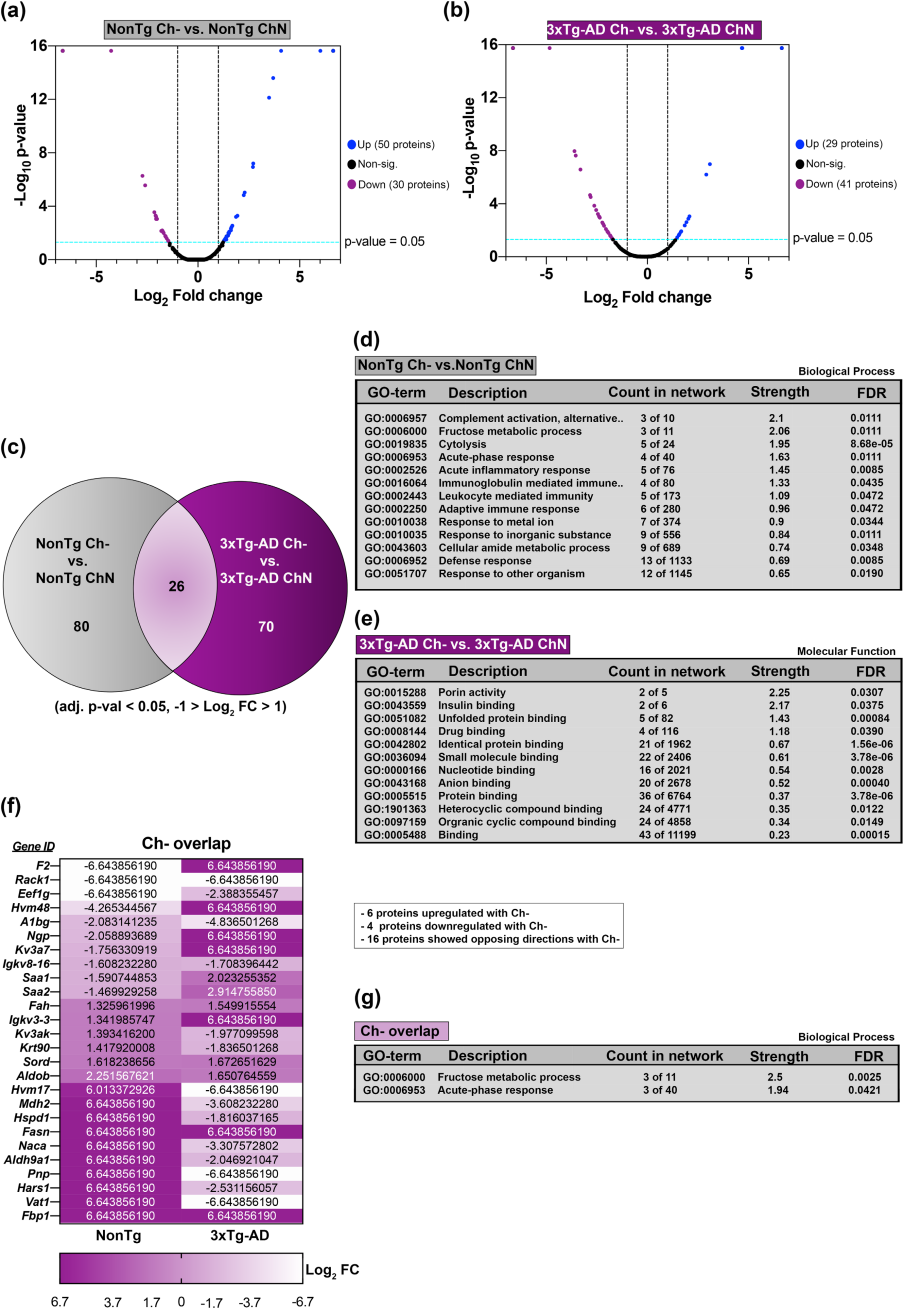
Ch‐ alters plasma protein networks in both NonTg and 3xTg‐AD mice. (a,b) Liquid chromatography‐tandem mass spectrometry followed by label‐free quantification (adj. *p*‐value < 0.05, −1 > Log_2_ FC > 1) identified 80 differentially abundant proteins between NonTg Ch‐ vs. NonTg ChN plasma, and 70 differentially abundant proteins between 3xTg‐AD ChN‐ vs. 3xTg‐AD ChN plasma. (c) Twenty‐six proteins were commonly identified as differentially abundant due to Ch‐ in both NonTg and 3xTg‐AD plasma. (d,e) NonTg Ch‐ vs. NonTg ChN gene ontology (GO) biological process classification analysis and 3xTg‐AD Ch‐ versus 3xTg‐AD ChN GO molecular function classification analysis. (f) List of genes and Log_2_ fold changes corresponding to the 26 proteins overlapping due to Ch‐. (h) Proteins overlapping due to Ch‐ GO biological process classification analysis

We next compared the plasma proteome of NonTg Ch‐ vs. NonTg ChN mice and the plasma proteome of 3xTg‐AD Ch‐ vs. 3xTg‐AD ChN mice (Figure S2). In the NonTg Ch‐ vs. NonTg ChN comparison, 80 differentially abundant proteins were identified. In the 3xTg‐AD Ch‐ vs. 3xTg‐AD ChN comparison, 70 differentially abundant proteins were identified (Figure 6a,b). Twenty‐six proteins were commonly identified as differentially abundant due to Ch‐ in both NonTg and 3xTg‐AD plasma (Figure 6c; Figure S2). GO of the list of differentially abundant plasma proteins in the NonTg Ch‐ vs. NonTg ChN comparison revealed dysregulation of biological processes related to inflammatory response, including acute‐phase response, acute inflammatory response, response to an inorganic substance, complement activation (alternative pathway), and defense response (Figure 6d; full GO Figure S3). Importantly, inflammation and immune dysfunction are relevant in a variety of diseases that affect all organs throughout the body, including AD (Husna Ibrahim et al., 2020; Kandimalla et al., 2017; Kneeman et al., 2012; Xie et al., 2022; Yang, Li, et al., 2020; Yang, Jiang, et al., 2020).

### 2.9 Ch‐ alters protein networks associated with insulin binding and porin activity in 3xTg‐AD plasma

Gene ontology for the list of differentially abundant plasma proteins in the 3xTg‐AD Ch‐ versus 3xTg‐AD ChN mice revealed that proteins associated with insulin binding were downregulated by Ch‐ (Figure 6e). This is consistent with the glucose metabolism dysfunction we identified in 3xTg‐AD Ch‐ mice, suggesting that Ch‐ may modulate insulin binding proteins in the plasma, and thereby contribute to glucose metabolism impairments. GO also revealed that proteins associated with porin activity were downregulated by Ch‐ (Figure 6e). These proteins and their roles in mitochondrial dysfunction have been previously associated with cell death and cognitive decline in AD patients (Shoshan‐Barmatz et al., 2018).

### 2.10 Ch‐ alters plasma protein networks associated with the fructose metabolic process and acute‐phase response in both NonTg and 3xTg‐AD mice

Of the 26 proteins whose levels were altered due to Ch‐ in both NonTg Ch‐ versus NonTg ChN and 3xTg‐AD Ch‐ versus 3xTg‐AD ChN comparisons (Figure 6f; Figure S2), 6 were upregulated, 4 were downregulated, and 16 were expressed in opposing directions. Notably, many of these proteins, such as Sord, Aldob, and F2, are produced in the liver (Stocks et al., 2022). GO revealed that this list of 26 proteins represented protein networks associated with the fructose metabolic process and acute‐phase response (Figure 6g). Fructose metabolism is central to obesity and NAFLD (Stocks et al., 2022), a phenotype observed in both NonTg and 3xTg‐AD Ch‐ mice. Additionally, the acute‐phase response is a reaction to systemic disturbances in homeostasis, which may be a result of metabolic differences seen with Ch‐ (Jang et al., 2019). Interestingly, both protein networks are consistent with the known role of choline in the human body, suggesting that Ch‐ driven changes in these proteins may be part of the mechanisms by which choline deficiency causes obesity, NAFLD, and metabolic dysfunction.

## 3 DISCUSSION

We find that dietary choline deficiency (Ch‐) throughout adulthood led to motor impairments, weight gain, impaired glucose metabolism, cardiac pathology, and liver disease, in both NonTg and 3xTg‐AD mice, and elevations of AD pathology in the 3xTg‐AD mouse. We confirmed significantly lower levels of choline in plasma and brain tissue with Ch‐ and found no compensation of liver PEMT for dietary choline deficiency. Notably, postmenopausal females and males do not respond to low choline supply due to a lack of PEMT induction, however, premenopausal females have been shown to be protected from low choline (Fischer et al., 2007). This is in part due to interactions between PEMT and estrogen (Fischer et al., 2007). Rodents do not become acyclic (have low estrogen) until 18–24 months, thus our mice, which were cycling throughout the study, were still unable to compensate for dietary Ch‐.

We observed motor deficits in the Ch‐ mice compared to the ChN counterparts. Choline is a precursor to acetylcholine, and there is a relationship between choline intake, acetylcholine, and motor function (Tabassum et al., 2017). It is important to note that Ch‐ mice did weigh significantly more than ChN animals, and heavier weight may be associated with the likelihood to fall off the spinning rod faster. 3xTg‐AD Ch‐ mice showed elevated Aβ pathology and tau phosphorylation in the Hp and Ctx, however, this elevation of AD pathology did not correspond to worse performance in the MWM. Our previous work has shown that adulthood choline supplementation, in similar‐aged mice, improved both AD pathology and cognitive performance in the MWM (Velazquez et al., 2019). Here, we hypothesize that further impairment of spatial learning and reference memory in the 3xTg‐AD Ch‐ mice did not occur because of ceiling effects in the MWM performance of the 3xTg‐AD ChN mice (Velazquez et al., 2019).

Hyperinflammation, mitochondrial dysfunction, and glucose metabolism impairments are observed in AD (Kandimalla et al., 2017; Velazquez et al., 2017). These dysfunctions are not unique to the brain and are seen across the entire body. Ch‐ mice had higher body weights than their ChN counterparts despite similar food consumption. Although 3xTg‐AD mice typically show glucose metabolism impairments relative to NonTg mice (Velazquez et al., 2017), 3xTg‐AD Ch‐ mice showed elevated glucose intolerance above that of their ChN counterparts. Notably, 3xTg‐AD mice ubiquitously express the PS1 M146V knockin mutation, while APPswe and MAPT P301L are predominantly expressed in the CNS (Velazquez et al., 2017). Since metabolic dysfunction was exacerbated in 3xTg‐AD Ch‐ mice, the ubiquitously expressed PS1 M146V knockin and Ch‐ may contribute to the dysregulated peripheral body defects in 3xTg‐AD mice. Dietary choline deficiency and PS1 knockin interactions should be investigated in future experiments. Further, NonTg Ch‐ mice also showed glucose metabolism impairments, compared to their ChN counterparts, and in fact, NonTg Ch‐ mice had similar dysfunction to that seen in 3xTg‐AD ChN mice. Choline plays an important role in energy metabolism (Zeisel, 2017), suggesting that Ch‐ can contribute to the development of a diabetic state. Type 2 diabetes mellitus (T2D) is a significant risk factor for AD (Kandimalla et al., 2017; Velazquez et al., 2017) and the two conditions are strongly interconnected (Kandimalla et al., 2017). 3xTg‐AD Ch‐ mice not only showed the most severe weight gain and glucose metabolism impairments but also had the highest pathological burden, suggesting that metabolism impairments may have contributed to exacerbated AD pathology.

Ch‐ has been linked to clinical markers of cardiovascular disease (Millard et al., 2018), and there is an established link between cardiovascular disease and the development of dementias, including AD (Yang, Li, et al., 2020; Yang, Jiang, et al., 2020). In the present study, we found that the 3xTg‐AD phenotype and the Ch‐ diet led to cardiac hypertrophy, with 3xTg‐AD Ch‐ mice showing the most severe cardiac hypertrophy. The expression of genes associated with cardiac pathology (*Col1a1*, *Myh7*, and *Nppa*) was elevated in all groups except the NonTg ChN mice, illustrating that Ch‐ was sufficient to induce cardiac hypertrophy in NonTg mice, and further exacerbate cardiac hypertrophy in 3xTg‐AD mice, collectively highlighting that Ch‐ may increase the risk for cardiac dysfunction.

Unsurprisingly, we detected markers of liver disease in Ch‐ mice; NonTg mice showed NAFLD as evidenced by hepatic steatosis, while 3xTg‐AD mice progressed to NASH, with evidence of inflammation and scarring alongside hepatic steatosis. Notably, while Ch‐ can induce NAFLD, work has shown that obesity, diabetes, and insulin resistance can also induce this phenotype, which was observed in both Ch‐ groups and may have also contributed to liver pathology (Kneeman et al., 2012). A healthy liver is critical for the clearance of circulating Aβ in the periphery and hepatic disease reduces the clearance of peripherally‐circulating Aβ (Estrada et al., 2019). The peripheral clearance of Aβ by the liver helps reduce the accumulation of Aβ into plaques (Estrada et al., 2019). We found that Aβ pathology was increased in the brains of 3xTg‐AD Ch‐ mice, and failure to effectively clear Aβ due to liver disease may partially contribute to the increased burden, although future work is necessary to further interrogate this hypothesis.

Proteomics analysis of Hp tissue identified Ch‐ induced changes in key proteins linked to AD‐related biological processes. Protein expression of Hp Mapt was upregulated in 3xTg‐AD ChN compared to NonTg ChN mice (Log_2_ FC = 0.97) and was downregulated (Log_2_ FC = −1.45) in 3xTg‐AD Ch‐ mice compared to the 3xTg‐AD ChN mice. This is notable given the important role of healthy tau in microtubule stabilization, protein transport, synaptic plasticity, and learning and memory (Velazquez et al., 2018). The proteomic analysis also revealed Ch‐ induced modulation of Hp protein networks associated with postsynaptic membrane regulation, suggesting that Ch‐ may also be linked to the synaptic dysfunction observed in AD. Interestingly Nptn, a critical protein for long‐term potentiation at Hp excitatory synapses, also linked to AD (Ilic et al., 2019), was downregulated in 3xTg‐AD ChN mice compared to NonTg ChN counterparts (Log_2_ FC = −1.55), and Ch‐ downregulated this protein in both NonTg (Log2 FC = −2.13) and 3xTg‐AD mice (Log_2_ FC = −3.44). Moreover, proteins that modulate AMPA receptors Cacng8 (Log_2_ FC = −1.00), Lrrtm1 (Log_2_ FC = −1.34), Frrs1l (Log_2_ FC = −1.38), as well as NMDA receptors Grin2a (Log_2_ FC = −1.49), Clstn1 (Log_2_ FC = −1.05), were also downregulated by Ch‐ (Bhouri et al., 2018; Roy et al., 2016; Stewart et al., 2019), suggesting that Ch‐ can contribute to postsynaptic membrane dysfunction in AD. Previous work has shown that choline modulates the expression of alpha7 nicotinic acetylcholine and Sigma‐1 receptors (Velazquez et al., 2019). Our results corroborate these previous findings that adulthood choline supplementation regulates the expression of postsynaptic receptors and suggest that choline alters postsynaptic receptor abundance through additional pathways outside of acetylcholine. Altogether, these results provide significant evidence that Ch‐ exacerbates AD pathology and synaptic dysfunction.

Given the systems‐wide dysfunction observed, we performed comparative proteomics on plasma and found that Ch‐ modulates key inflammatory, immune response, acute‐phase response, and fructose metabolic process pathways. Within the acute inflammatory and phase response pathways, 3xTg‐AD ChN mice show downregulation of Saa1 (Log_2_ FC = −2.45) and Saa2 (Log_2_ FC = −2.51) compared to NonTg counterparts. Ch‐ upregulated Saa1 (Log_2_ FC = 2.02) and Saa2 (Log_2_ FC = 2.91) in 3xTg‐AD mice, but downregulated Saa1 (Log_2_ FC = −1.59) and Saa2 (Log_2_ FC = −1.47) in NonTg mice. These two serum amyloid proteins are produced peripherally as an inflammatory response to environmental insults (Jang et al., 2019). Saa1 has been shown to prime microglia for ATP‐dependent interleukin‐1B release, which is associated with AD onset (Jang et al., 2019). These findings are particularly insightful, as they corroborate previous literature that adulthood choline supplementation in an AD mouse model decreases disease‐associated microglial activation (Velazquez et al., 2019). Previous literature also indicates that glial cell populations are responsive to Saa1 secretion in the blood and that Saa1 overexpression increases amyloid aggregation and glial activation in an AD mouse model (Jang et al., 2019). This suggests that Ch‐ modulates Saa1/2 secretion in the plasma, causing glial hyperactivation, increased neuroinflammation, and potentially increased Aβ aggregation. Pertaining to fructose metabolic processing, Ch‐ led to upregulation of Aldob and Sord in both NonTg (Log_2_ FC = 2.25, 1.62) and 3xTg‐AD (Log_2_ FC = 1.65, 1.67) mice, which are both produced in the liver. Obesity and liver pathology have been linked to fructose metabolic processing and an upregulation of Aldob (Stocks et al., 2022), suggesting that Ch‐ may induce obesity and liver pathology phenotypes, leading to the upregulation of these proteins. Relative to glucose metabolism impairments caused by Ch‐ in healthy aging and AD, we find that a plasma protein network associated with insulin binding (containing insulin‐degrading enzyme (Ide) and heat shock protein family D (Hspd1, also known as Hsp60)) was altered due to Ch‐ in 3xTg‐AD mice (Log_2_ FC = −1.92, −1.81, respectively). Notably, both ide (Log_2_ FC = 2.57) and Hspd1 (Log_2_ FC = 6.64) were upregulated in 3xTg‐AD ChN mice compared to NonTg ChN. Ide degrades insulin, is directly related to insulin resistance, and also degrades Aβ (Kandimalla et al., 2017). Ide and its impact on insulin resistance have been linked to cognitive impairment (Yang, Li, et al., 2020; Yang, Jiang, et al., 2020). Additionally, there is growing evidence that Hspd1 modulates diabetes‐induced inflammation and protects against Aβ oligomer‐induced synaptic toxicity (Liyanagamage & Martinus, 2020; Marino et al., 2019). This is particularly interesting given the large body of literature that demonstrates the negative effects of T2D on brain insulin resistance, oxidative stress, and cognitive decline (Kandimalla et al., 2017). Taken together, Ch‐ modulates key insulin‐binding proteins, which may contribute to the diabetes‐like pathology observed in AD cases. Finally, mitochondrial dysfunction has emerged as another driver of AD pathology (Kandimalla et al., 2017). Our results show that 3xTg‐AD ChN mice exhibit upregulation of two plasma proteins involved in porin binding, Vdac1 (Log_2_ FC = 2.38) and Vdac2 (Log_2_ FC = 6.64) compared to NonTg ChN mice, consistent with literature showing increases in AD models (Shoshan‐Barmatz et al., 2018). Ch‐ in 3xTg‐AD mice downregulates Vdac1 (Log_2_ FC = −6.64) and Vdac2 (Log_2_ FC = −2.84): these two proteins have also been linked to mitochondrial dysfunction and AD pathology (Shoshan‐Barmatz et al., 2018; Singulani et al., 2020). This suggests that Vdac1 and Vdac2 modulation by Ch‐ may induce mitochondrial dysfunction in AD mouse models. Vdac1 has been shown to mediate Aβ toxicity in the brain, and its overexpression triggers cell death (Shoshan‐Barmatz et al., 2018). Additionally, Vdac1 interacts with several AD‐relevant proteins, including pTau, Aβ, and gamma‐secretase (Shoshan‐Barmatz et al., 2018). Vdac2 has been shown to decrease with age and with increased pathology in 3xTg‐AD, suggesting that Ch‐ may downregulate this protein, contributing to mitochondrial dysfunction (Singulani et al., 2020).

In conclusion, adequate choline intake is important for health across a variety of bodily systems; metabolic, cardiac, liver, and neurological. If generalized to humans, these findings may help mitigate the estimated increase in the prevalence of AD and illustrate the importance of adequate dietary choline intake throughout adulthood to offset disease occurrence for the general population.

## 4 MATERIALS AND METHODS

### 4.1 Animals and study design

3xTg‐AD homozygous mice were generated as previously described (Velazquez et al., 2017). C57BL6/129Svj mice were used as non‐transgenic controls (NonTg). Only female 3xTg‐AD mice were used because males do not display consistent neuropathology (Velazquez et al., 2017). Mice were kept on a 12‐h light/dark cycle at 23°C with ad libitum access to food and water and group‐housed, four to five per cage. Mice were randomly assigned to one of two diets at 3 months of age; a standard laboratory AIN76A diet (Envigo Teklab Diets, Madison WI) with normal choline levels (ChN; 2.0 g/kg; #TD.180228), based on the human ADI or an AIN76A choline‐deficient (Ch‐; 0.0 g/kg; #TD.110617) diet. Diet start age was selected due to choline's important role in the developing brain (Blusztajn, 1998). All animal procedures were approved in advance by the Institutional Animal Care and Use Committee of Arizona State University.

### 4.2 Behavioral testing

At 10 months of age, mice underwent 3 days of rotarod testing (AccuScan Instruments Inc.) to assess motor ability as previously described (Velazquez et al., 2018). Mice received 6 trials/day for 3 days. During the two training days, a rod increased by 0.75 rpm/s over 20 s for a maximum speed of 15 rpm, and then remained constant at 15 rpm for the remaining 70 s. On the probe day, the rod accelerated at a steady increase of 1 rpm/s for up to 90 s. Mice were then tested in the Morris water maze (MWM), to assess hippocampal‐dependent spatial learning and memory, as previously described (Velazquez et al., 2019, 2020). All mice underwent 4 trials/day/5 days for training. The location of the hidden platform remained constant, but the start location pseudo‐randomly varied across trials. Mice were given 60 s/trial to locate the hidden platform. Twenty‐four hours after the last training session, the platform was removed, and mice were returned to the MWM for 60 s to assess spatial reference memory. Data were analyzed via EthoVisionXT (Noldus Information Technology).

### 4.3 Weight, food consumption, and glucose tolerance (GTT) test

Weight was measured biweekly. A food consumption test was performed in 9‐month‐old mice as previously described (Ellacott et al., 2010). Mice were housed in 18 cages balanced for genotype and diet. Food was added in equal amounts on Day 1 and weighed every 24 h for 6 days to assess intake, measuring food consumed per day/number of mice in a cage. A GTT was performed as previously described prior to euthanasia (Velazquez et al., 2017). Animals were fasted overnight for 16 h, and baseline fasting glucose levels were taken. Animals received a 2.0 mg/kg glucose intraperitoneal (i.p.) injection and blood glucose was sampled from the tail using a TRUEtrack glucose meter and TRUEtrack test strips (Trividia Health) at 15, 30, 45, 60, 90, 120, and 150 min following the injection.

### 4.4 Blood collection and plasma extraction

Mice were fasted for 16 h, and blood was collected via the submandibular vein. 150–200 μl (≤10% of the subject's body weight) of blood was collected and placed into EDTA‐lined tubes (BD K_2_EDTA #365974) and inverted eight times to assure anticoagulation. Tubes were kept on ice for 60–90 min and then centrifuged at 2200 RPM for 30 min at 4°C to separate phases. The top layer was collected and frozen at −80°C.

### 4.5 Tissue harvesting and processing

Mice were euthanized at an average of 12 months of age. One set of mice was perfused with 1X PBS and had brains and livers extracted and fixed in a glass vial filled with 4% paraformaldehyde for 48 h. The remaining non‐perfused mice had their Hp and Ctx tissue dissected and prepared for protein assays as previously described (Velazquez et al., 2019, 2017). Hearts were removed, weighed, and immediately flash‐frozen in dry ice. Heart total RNA was extracted from the left ventricular extract using the RNeasy Mini Kit (Qiagen) as previously described (Blackwood et al., 2019). All qPCR probes were obtained from Integrated DNA Technologies.

### 4.6 ELISA, western blots, and choline assays

We used commercially available ELISA kits (Invitrogen‐ThermoFisher Scientific) to quantify soluble and insoluble levels of Aβ, and levels of pTau at Ser181 and Ser396 as previously described (Velazquez et al., 2019, 2017). Choline levels were quantified using commercially available kits (Abcam, ab219944). Western blots were performed under reducing conditions as previously detailed (Velazquez et al., 2017) to probe for PEMT (dilution 1:1000, Thermo Fisher Scientific, PA5‐42383) and loading control GAPDH (dilution 1:5000, Abcam ab8245).

### 4.7 Tissue sectioning and histology

Liver fixed tissue was sectioned at 20 μm using a vibratome (Leica VT1000S), stained using a Hematoxylin and Eosin (H&E) kit (Abcam, ab245880), and imaged on a light microscope at 40X (Zeiss Axio Imager M2) to score for pathology as previously described (Liang et al., 2014). Brain hemispheres were sectioned into 50 μm coronal sections using a vibratome and stored in specimen plates in PBS with 0.02% sodium azide. For Thioflavin S staining, tissue sections were incubated in 4% paraformaldehyde for 15 min, filtered 1% aqueous Thioflavin S for 10 min at room temperature, washed twice in 80% ethanol, once in 95% ethanol, and 3 times in ddH2O. Images were taken at 5× on a fluorescence microscope (Leica Thunder) and quantified using Image J for structure number. AT8 immunohistochemistry was performed as previously described (Dave et al., 2021) with the appropriate antibodies (1:1000 dilution, Thermo Fisher Scientific, catalog# MN1020).

### 4.8 Unbiased stereology

Stereoinvestigator 17‐software (Micro‐BrightField, Cochester, VT) optical fractionator method was used to quantify AT8‐positive CA1 cells in the Hp as previously described (Dave et al., 2021). Counts were performed at predetermined intervals; Grid size (*X* and *Y* = 158 μm), counting frame (*X* and *Y* = 50 μm), superimposed on the live image of the tissue sections. The sections were analyzed using a 63*x* × 1.4 PlanApo oil immersion objective. Gunderson's score remained ≤0.08. The average tissue thickness was 26 μm. Dissector height was set at 22 μm, with a 2‐μm top and 2‐μm bottom guard zone. Seven sections were evaluated per animal. Bright‐field photomicrographs were taken with a Zeiss Axio Imager. The AT8 antibody penetrated the full depth of the section, allowing for an equal probability of counting all objects.

### 4.9 Liquid chromatography‐tandem mass spectrometry (LC–MS/MS)

For LC–MS/MS, solubilized Hp mouse brain and plasma proteins were quantified (Thermo Fisher EZQ Protein Quantitation Kit or the Pierce BCA). Proteins were reduced with 50 mM dithiothreitol (Sigma‐Aldrich) final concentration at 95°C for 10 min and alkylated with iodoacetamide (Pierce,40 mM final 30 min). Proteins were digested using 2.0 μg of MS‐grade porcine trypsin (Pierce) and peptides were recovered using Protifi S‐trap Micro Columns per manufacturer directions. Recovered peptides were dried via speed vac and resuspended in 30 μl of 0.1% formic acid.

All data‐dependent mass spectra were collected in positive mode using an Orbitrap Fusion Lumos mass spectrometer (Thermo Scientific) coupled with an UltiMate 3000 UHPLC (Thermo Scientific). One μL of the peptide was fractionated using an Easy‐Spray LC column (50 cm × 75 μm ID, PepMap C18, 2 μm particles, 100 Å pore size, Thermo Scientific) with an upstream 300 μm × 5 mm trap column. Electrospray potential was set to 1.6 kV and the ion transfer tube temperature to 300°C. The mass spectra were collected using the “Universal” method optimized for peptide analysis provided by Thermo Scientific. Full MS scans (375–1500 m/z range) were acquired in profile mode with the following settings: Orbitrap resolution 120,000 (at 200 m/z), cycle time 3 seconds and mass range “Normal;” RF lens at 30% and the AGC set to “Standard”; maximum ion accumulation set to “Auto;” monoisotopic peak determination (MIPS) at “peptide” and included charge states 2–7; dynamic exclusion at 60 s, mass tolerance 10 ppm, intensity threshold at 5.0e^3^; MS/MS spectra acquired in a centroid mode using quadrupole isolation at 1.6 (m/z); collision‐induced fragmentation (CID) energy at 35%, activation time 10 ms. Spectra were acquired over a 240‐min gradient, flow rate 0.250 μl/min as follows: 0–3 min at 2%, 3–75 min at 2%–15%, 75–180 min at 15%–30%, 180–220 min at 30%–35%, 220–225 min at 35%–80% 225–230 at 80% and 230–240 at 80–5%.

### 4.10 Label‐free quantification (LFQ) and gene ontology (GO)

Raw spectra were loaded into Proteome Discover 2.4 (Thermo Scientific) and protein abundances were determined using the Uniprot (www.uniprot.org) *Mus musculus* database (Mmus_UP000000589.fasta). Protein abundances were determined using raw files and were searched using the following parameters: Trypsin as an enzyme, maximum missed cleavage site 3, min/max peptide length 6/144, precursor ion (MS1) mass tolerance at 20 ppm, fragment mass tolerance at 0.5 Da, and a minimum of 1 peptide identified. Carbamidomethyl (C) was specified as fixed modification, and dynamic modifications set to Acetyl and Met‐loss at the N‐terminus, and oxidation of Met. A concatenated target/decoy strategy and a false‐discovery rate (FDR) set to 1.0% were calculated using Percolator. Accurate mass and retention time of detected ions (features) using the Minora Feature Detector algorithm were then used to determine the area‐under‐the‐curve (AUC) of the selected ion chromatograms of the aligned features across all runs and the relative abundances calculated. GO analyses for biological process classification and molecular function classification were performed using STRINGv11.5 (Search Tool for the Retrieval of Interacting Genes/Proteins) as previously described (Szklarczyk et al., 2019).

### 4.11 Statistical analyses

Two‐way factorial Analysis of variance (ANOVA; for genotype and diet) was used to analyze the physiological and pathological experiments, followed by Bonferroni's corrected post hoc tests when appropriate. Repeated measures ANOVA was used to analyze the behavior and GTT data. Student's unpaired t‐tests were utilized for comparison of 3xTg‐AD Ch‐ vs. ChN mice. Levene's test for homogeneity of variance revealed no significant effects. Significance was set at *p* < 0.05.

## AUTHOR CONTRIBUTIONS

ND and JMJ: Equal contribution to writing, data analysis, and animal experiments. AD: Animal studies, histology, and edited the manuscript. WW: ELISA, statistical analysis, and wrote the manuscript. PS: Liver pathology assessment and wrote the manuscript. OVE: Liver pathology assessment and wrote the manuscript. ST: Animal studies, figure creation, and edited the manuscript. SKB: Histology, data analysis, and edited the manuscript. AB: Cardiac assessment and edited the manuscript. JS: unbiased proteomics and edited the manuscript. IM: Data analysis and edited the manuscript. JKW: Data analysis and edited the manuscript. EAB: Cardiac assessment and edited the manuscript. CG: Experimental design and cardiac assessment. TM: Proteomic analysis and wrote the manuscript. RV: Experimental design, animal studies, and wrote the manuscript. All authors read and approved the final manuscript.

## CONFLICT OF INTEREST

The authors have declared that no conflict of interest exists.

## Supporting information


FigureS1

FigureS2

FigureS3

Tables

## Data Availability

Proteomic data have been deposited to the ProteomeXchange Consortium via the PRIDE partner repository. The data that support the findings of this study are available from the corresponding author, R.V., upon reasonable request.
