# Supplementary figures and images for: Dietary choline intake is necessary to prevent systems‐wide organ pathology and reduce Alzheimer's disease hallmarks
Source: Aging Cell. 2023 Jan 15;22(2):e13775. doi: 10.1111/acel.13775 (PMC9924938; doi:10.1111/acel.13775)

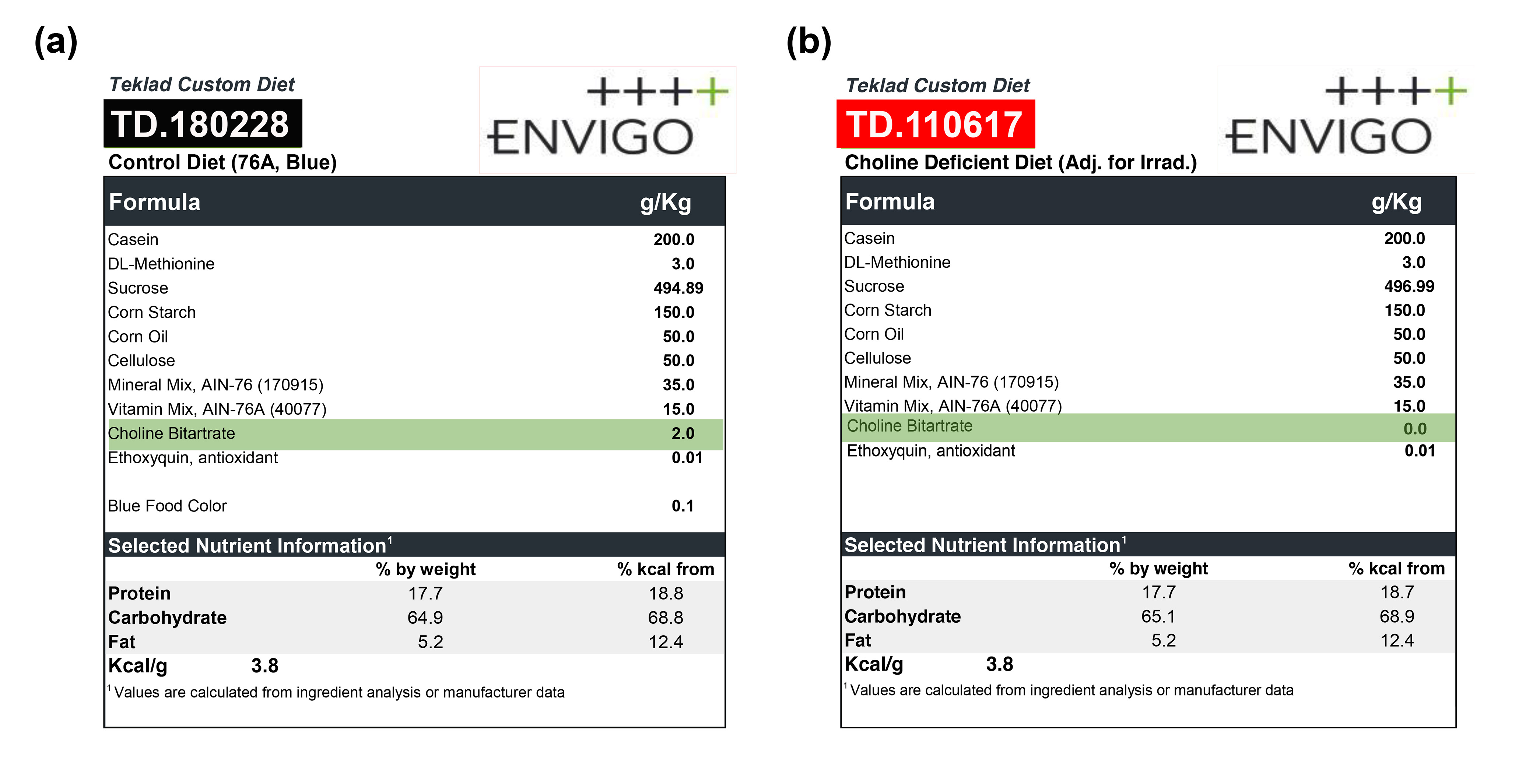

Supplement: Supplementary file 1 — FigureS1 [file ACEL-22-e13775-s004.jpg]
