# Supplementary material for: Dietary choline intake is necessary to prevent systems‐wide organ pathology and reduce Alzheimer's disease hallmarks
Source: Aging Cell. 2023 Jan 15;22(2):e13775. doi: 10.1111/acel.13775 (PMC9924938; doi:10.1111/acel.13775)
Supplement: Supplementary file 4 — Tables [file ACEL-22-e13775-s002.docx]

| ***Gene***  ***symbol*** | **Description** | **Log_2_ FC (3xTg-AD ChN / NonTg ChN)** | **Abundance Adj.**  **-Log_10_ p-value** |
| --- | --- | --- | --- |
| *Nptn* | Isoform 3 of Neuroplastin | -1.55215636 | 17.00000000 |
| *Smdt1* | Essential MCU regulator mitochondrial | -1.53951953 | 10.20483336 |
| *Apoa1* | Apolipoprotein A-I | -1.27229733 | 13.82105086 |
| *App* | Amyloid-beta A4 protein | 1.58448152 | 17.00000000 |
| *Hdac1* | histone deacetylase 1 | 2.15704371 | 12.59513375 |
| *Mapt* | Microtubule-associated protein tau | 0.9701174 | 12.73448168 |

**Supplementary Table 1.** Significant Alzheimer’s disease related hippocampal (Hp) proteins in 3xTg-AD ChN vs. NonTg ChN comparison (n = 4 mice / genotype). Purple indicates downregulated in 3xTg-AD ChN, blue is upregulated in 3xTg-AD ChN.

| ***Gene***  ***symbol*** | **Description** | **Log_2_ FC (3xTg-AD ChN / NonTg ChN)** | **Abundance Adj. --Log_10_ p-value** |
| --- | --- | --- | --- |
| *Saa2* | Serum amyloid A-2 protein | -2.514573173 | 3.20994107 |
| *Saa1* | Serum amyloid A-1 protein | -2.450084446 | 3.05193239 |
| *Vdac1* | Voltage-dependent anion-selective channel protein 1 | 2.384602458 | 2.67155179 |
| *Ide* | Insulin-degrading enzyme | 2.566815154 | 3.15824222 |
| *Vdac2* | Outer mitochondrial membrane protein porin 2 | 6.64385619 | 15.95326866 |
| *Hspd1* | 60 kDa heat shock protein, mitochondrial (Fragment) | 6.64385619 | 15.95326866 |

**Supplementary Table 2.** Significant Alzheimer’s disease related plasma proteins in 3xTg-AD ChN vs. NonTg ChN comparison (n = 4 mice / genotype). Purple indicates downregulated in 3xTg-AD ChN, blue is upregulated in 3xTg-AD ChN.
